# Asymmetry of interlimb transfer: Pedagogical innovations in physical education

**DOI:** 10.3389/fpsyg.2022.1029888

**Published:** 2022-11-07

**Authors:** Qun Fang, Yuhang Xia, Xiaochao Zhang, Fang Huang

**Affiliations:** School of Physical Education, Qingdao University, Qingdao, China

**Keywords:** interlimb transfer, asymmetry, hemispheric lateralization, physical education, pedagogical innovation

## Introduction

Interlimb transfer has long been identified in motor learning, with the consensus that the first research can be traced back to 1894 (Scripture et al., 1894). Effects of unilateral practice transfer to the contralateral limb, resulting in skill improvement of the untrained side (Green and Gabriel, 2018). Research evidence based on the neuroimaging technique suggests that interlimb transfer is more than a phenomenon at the behavioral level. In fact, it is associated with strengthened neural correlates within the motor network (Dirren et al., 2021). A noticeable feature of interlimb transfer is the asymmetrical pattern which indicates greater transfer in one direction than that in the other (Wang and Sainburg, 2004, 2006). Asymmetry of interlimb transfer has been well-documented in a number of motor learning research on manual performance (Sainburg and Wang, 2002; Lavrysen et al., 2003; Teixeira and Caminha, 2003; Galea et al., 2007). The typical design induces learning experience during the experiment by means of visuomotor rotations (Wang and Sainburg, 2004, 2006). Participants initially performed an aiming task by moving a cursor from a start point to a target over a tablet. The cursor displayed the location of the finger directing the cursor. After practices under normal circumstances, visual perturbations were assigned by 30° counterclockwise rotation. In the visuomotor rotation experiment, motor learning represents conscious and progressive corrections for perceived movement errors due to the distorted visual feedback (Taylor et al., 2014).

Theories on the asymmetrical interlimb transfer have been developing over the past decades. The proficiency model is an early hypothesis to provide insights into the asymmetrical transfer. Dominant hemisphere/hand is more proficient than non-dominant hemisphere/hand in motor learning. Practice on the dominant side results in more motor information available for transfer to the non-dominant side. Therefore, the model suggests greater transfer to the non-dominant limb performance following the dominant limb practice (Laszlo et al., 1970). On the other hand, the callosal access model predicts an opposite transfer direction which favors the dominant limb performance (Taylor and Heilman, 1980). According to this model, motor information is stored in the dominant hemisphere, thus facilitating learning and performance of the dominant limb due to the direct access to the stored information. While interpreting the asymmetrical transfer to some extent, the two models still show major limitations as each model only covers one direction of interlimb transfer. Based on critical analysis of the limitations, Parlow and Kinsbourne (1989) proposed cross activation model with a particular emphasis on bilateral adaptations. This model is based on the observation that unilateral movement activates both contralateral and ipsilateral motor cortices (Dai et al., 2001; van Duinen et al., 2008). Unilateral training over time leads to neural adaptations in both hemispheres, and the neural adaptations in the untrained hemisphere enhance performance of the untrained limb (Ruddy and Carson, 2013).

The limitations of previous models called for more inclusive perspectives on the asymmetrical transfer in motor learning. Sainburg and Wang (2002) provided evidence that transfer direction was specific to task features. In the same arm reaching task, opposite transfer directions were observed in different kinematic measures. Greater contralateral transfer was identified in the initial direction of right arm movements under the rotated visual conditions. On the other hand, right arm training resulted in greater improvement in the final position accuracy of left arm movements. The findings suggest complex mechanisms underlying interlimb transfer, leading to further investigations on the functional specialization in left and right hemispheres.

Dynamic-dominance hypothesis assumes distinct neural mechanisms for particular movement features (Bagesteiro and Sainburg, 2002). While the dominant system is responsible for the regulation of dynamic characteristics of movement (i.e., control of trajectories and force production), the non-dominant system processes visual-spatial information of movement (i.e., control of final positions and targeted precision) (Sainburg, 2002; Stöckel and Weigelt, 2012). Hemispheric specialization for different motor control mechanisms has been shown to influence directions and magnitude of interlimb transfer (Sainburg et al., 2016). With the increasing understandings of hemispheric lateralization and interlimb transfer, the model of specialized processing and transfer was proposed with an essential hypothesis that practice involving the specialized hemisphere-effector system induces a larger transfer of learning than the non-specialized, less efficient hemisphere-effector system (Stöckel et al., 2011). Recent research provided evidence for the model of specialized processing and transfer. In a grooved pegboard task, right-handed participants showed greater transfer after right hand practice than that after left hand practice whereas left-handed participants performed the task with comparable magnitude of transfer after practice on each hand (Wang et al., 2020). Handedness is reflective of hemispheric asymmetry. The different transfer directions between right- and left-handed individuals suggest the impact of hemispheric asymmetry on interlimb transfer. Additionally, hemispheric activations tend to become less asymmetrical in response to increased task complexity. Asymmetrical transfer has been reported in simple tasks during which hemispheric activation is lateralized. On the other hand, complex tasks stimulate bilateral activations, leading to symmetrical interlimb transfer associated with the reduced hemispheric lateralization (Wang et al., 2022). Therefore, the findings of handedness and task complexity suggest that the model of specialized processing and transfer can provide valid interpretations on asymmetry of interlimb transfer during motor learning.

Motor learning research has been conducted to expand understandings of the neural mechanisms. However, existing research with the purpose of applying neuroscience knowledge and principles to enhance motor learning is still limited. An interesting speculation can be reached about the feasibility to enhance teaching and learning in physical education (PE) by taking advantage of the asymmetrical phenomenon in interlimb transfer. So far, interlimb transfer has been mainly investigated by simple motor actions such as finger tapping and pegboard tasks. It is reasonable to examine whether the asymmetrical pattern observed in the laboratory settings can be extended to the practical conditions in PE classes. The current study proposed the opinion that asymmetry of interlimb transfer may contribute to pedagogical innovations in PE by facilitating the process of teaching and learning sport skills. PE class is the primary affordance for students to learn a novel sport skill (Stöckel et al., 2011). Research on asymmetrical interlimb transfer implies practical value in developing effective teaching strategies to enhance sport skill acquisition.

## Pedagogical innovations in PE

Asymmetry of interlimb transfer is indicative of hemispheric functioning. Neuroimaging evidence has shown adaptations in neural circuits associated with improved performance of the untrained limb after unilateral training (Oosawa et al., 2019). Childhood and adolescence are critical periods during which the nervous system is highly plastic (Shaw et al., 2006; Ismail et al., 2017). PE class design in consideration of neural functioning may enhance sport skill acquisition. In recent years, neuroscience is characterized by prominent progress in improving the teaching and learning process for many subjects (Baena-Extremera et al., 2021). Research on asymmetrical interlimb transfer may provide a promising approach to integrate neuroscience into class design and organization, leading to pedagogical innovations in the field of PE.

## Class design based on asymmetrical interlimb transfer

While improved performance of the trained limb indicates the principle of specificity, it is also necessary to notice the transfer effects which enhance performance of the untrained limb. Because interlimb transfer is asymmetrical in direction and magnitude, PE teachers and practitioners should consider practicing the side that results in larger transfer effects. For a specific sport skill, if the right limb practice benefits both right and left limb performance whereas the left limb practice only produces positive effect on the left limb performance, a reasonable selection is to practice the skill with emphasis on the right side. Haaland and Hoff (2003) designed a soccer training program in which the experimental group performed skill practice by non-dominant (left) foot and the control group practiced with no particular demand on which side to use. The experimental group enhanced both left and right foot performance, whereas the control group only improved right foot performance. Between-group comparisons indicated that practice on the non-dominant foot resulted in superior left foot performance and comparable right foot performance to the control group. The findings warrant implementing non-dominant foot practice in acquisition of basic soccer skills.

Whether dominant or non-dominant limb practice produces greater transfer depends on the inherent motor components of the task (Sainburg and Wang, 2002). For the skills involving the visual-spatial information which specializes in the right hemisphere/left limb, particular emphasis on the left limb should be given during the practice. Additionally, dynamic characteristics of movement are processed in the left hemisphere/right limb system. Greater transfer effects can be achieved by the right limb practice on the skills involving the regulation of movement dynamics.

Evidence in accordance with the hypothesis was provided in a study which examined two types of throwing skills with particular demands on accuracy and force production (Stöckel and Weigelt, 2012). The training program lasted 8 weeks during which participants began the training with one hand in the first 4 weeks and then switched to the other hand practice in the last 4 weeks. In the task with an emphasis on throwing accuracy, initial practice on the non-dominant hand enhanced performance of both hands to a greater extent than the opposite order. In contrast, participants with initial practice on the dominant arm showed larger beneficial transfer than their counterparts with initial practice on the non-dominant arm when performing the throwing task with an emphasis on maximum force.

The throwing tasks with a particular demand on accuracy involves visual-spatial processing (e.g., trajectory control) in the right hemisphere/left limb system. Practice on the left hand is in line with the specialized hemisphere-effector system, thus resulting in greater transfer effects. Same rules can be applied to the task with a demand on throwing force. The left hemisphere/right hand system specializes in regulation of movement dynamics such as force production (Serrien et al., 2006). Practice on the dominant hand establishes a better representation of the specialized left hemisphere/right hand system in motor skill acquisition, leading to larger transfer from the right hand practice to the left hand performance.

Additional evidence was provided by 6-week fencing training sessions which were developed in accordance with the hemispheric lateralization hypothesis (Witkowski et al., 2018, 2020). The ratio of non-dominant (left) side to dominant (right) side practice was 3:1, as each drill repeated three times on the left limb associated with one practice on the right limb. The control group, on the other hand, implemented practice only with the dominant side. Although fencing is a unilateral sport, bilateral practice resulted in greater improvement in hitting accuracy of the dominant hand than that after unilateral practice. Because the right hemisphere/left limb system specializes in spatial characteristics of movement, training with an emphasis on the left side enhances the specific neural network, producing transfer effects to the dominant hand performance. Therefore, evidence from the existing research implies promising applications of asymmetrical transfer to PE. Class design in accordance with asymmetry of interlimb transfer would promote time efficiency of teaching and learning.

## Framework for future endeavors

Despite the promising applications of interlimb transfer, direct evidence with respect to pedagogical practice is still limited, which warrants future research on the relevant topic. A four-phase framework to guide subsequent research work is provided in this section.

Successful application of asymmetrical interlimb transfer to PE underlies the link between the trained limb and the specialized hemisphere-effector system in skill learning. The initial phase should classify individual sport skills (e.g., passing and dribbling) into corresponding hemisphere-effector systems. The fundamental work is to identify the skills which are dominantly processed by the right hemisphere/left limb system and those mainly processed by the left hemisphere/right limb system. The second phase involves conducting experimental studies to testify efficacy of the existing models in predicting and explaining the effects of applying asymmetrical transfer to sport skill acquisition. Marinsek (2016) provided a valid study design to examine lateral asymmetry in dribbling skill practice. Participants were randomly allocated to dominant limb practice group, non-dominant limb practice group, or bilateral practice group. Outcomes in relation to each teaching strategy were examined by within- and between-group comparisons. The third phase, after the study design and implementation, aims to interpret the results by available theoretical models. The findings may be inconsistent with the hypothesis that practice on the specialized hemisphere-effector system induces a better transfer of learning across limbs. Indeed, inconsistent findings have been reported by the existing literature. For the dribbling practice in soccer, some studies identified better learning outcomes associated with non-dominant limb practice (Haaland and Hoff, 2003; Teixeira et al., 2003), while evidence also indicated favorable transfer effects after dominant limb practice (Marinsek, 2016). The magnitude and direction of transfer may be influenced by various factors such as age of participants (Marinsek, 2016) and types of sport skills (Stöckel and Weigelt, 2012). It is possible that the existing models may be inadequate to interpret all research findings, thus leading to the fourth phase of refining the models if necessary. With the cumulative understandings of the mechanisms underlying interlimb transfer in sport skill acquisition, principles of neuroscience and motor control can be better applied to enhance teaching and learning effects in PE classes.

## Conclusion

The current study highlighted asymmetry of interlimb transfer as a potential contribution to pedagogical innovations in the field of PE. Asymmetrical transfer is considered a reflection of functional lateralization in left and right hemispheres. Taking advantage of this interlimb phenomenon may enhance time efficiency of teaching and learning a novel sport skill. The basic rule to guide teaching practice can be summarized in the statement that practice involving the specialized hemisphere-effector system induces greater transfer effects, thus facilitating acquisition of sport skills. The empirical evidence implies high practical value for PE class design and implementation, which warrants future research work on the relevant topic. Accordingly, a four-phase framework was proposed to guide following research and pedagogical practice. The current study calls for more attention to the innovative teaching strategy which indicates a promising combination between neuroscience and PE.

## Author contributions

QF and YX prepared the draft. XZ and FH worked on revision and approved the submitted version. All authors collaborated in preparing the manuscript. All authors contributed to the article and approved the submitted version.

## Conflict of interest

The authors declare that the research was conducted in the absence of any commercial or financial relationships that could be construed as a potential conflict of interest.

## Publisher's note

All claims expressed in this article are solely those of the authors and do not necessarily represent those of their affiliated organizations, or those of the publisher, the editors and the reviewers. Any product that may be evaluated in this article, or claim that may be made by its manufacturer, is not guaranteed or endorsed by the publisher.

## References

[B1] Baena-Extremera A. Ruiz-Montero P. J. Hortigüela-Alcalá D. (2021). Neuroeducation, motivation, and physical activity in students of physical education. Int. J. Environ. Res. Public Health 18, 2622. 10.3390/ijerph18052622 33807889 PMC7967370

[B2] Bagesteiro L. B. Sainburg R. L. (2002). Handedness: dominant arm advantages in control of limb dynamics. J. Neurophysiol. 88, 2408–2421. 10.1152/jn.00901.2001 12424282 PMC10709816

[B3] Dai T. H. Liu J. Z. Sahgal V. Brown R. W. Yue G. H. (2001). Relationship between muscle output and functional MRI-measured brain activation. Exp. Brain Res. 140, 290–300. 10.1007/s002210100815 11681304

[B4] Dirren E. Bourgeois A. Klug J. Kleinschmidt A. van Assche M. Carrera E. (2021). The neural correlates of intermanual transfer. Neuroimage. 15, 118657. 10.1016/j.neuroimage.2021.118657 34687859

[B5] Galea J. M. Miall R. C. Woolley D. G. (2007). Asymmetric interlimb transfer of concurrent adaptation to opposing dynamic forces. Exp. Brain Res. 182, 267–273. 10.1007/s00221-007-1069-y 17703286 PMC3032218

[B6] Green L. A. Gabriel D. A. (2018). The cross education of strength and skill following unilateral strength training in the upper and lower limbs. J. Neurophysiol. 120, 468–479. 10.1152/jn.00116.2018 29668382 PMC6139459

[B7] Haaland E. Hoff J. (2003). Non-dominant leg training improves the bilateral motor performance of soccer players. Scand. J. Med. Sci. Sports 13, 179–184. 10.1034/j.1600-0838.2003.00296.x 12753491

[B8] Ismail F. Y. Fatemi A. Johnston M. V. (2017). Cerebral plasticity: windows of opportunity in the developing brain. Eur. J. Paediatr. Neurol. 21, 23–48. 10.1016/j.ejpn.2016.07.007 27567276

[B9] Laszlo J. I. Baguley R. A. Bairstow P. J. (1970). Bilateral transfer in tapping skill in the absence of peripheral information. J. Mot. Behav. 2, 261–271. 10.1080/00222895.1970.10734884 23941320

[B10] Lavrysen A. Helsen W. F. Tremblay L. Elliott D. Adam J. J. Feys P.. (2003). The control of sequential aiming movements: the influence of practice and manual asymmetries on the one-target advantage. Cortex 39, 307–325. 10.1016/S0010-9452(08)70111-4 12784891

[B11] Marinsek M. (2016). Lateral asymmetry as a function of motor practice type of complex upper- and lower-limb movement in young children. Laterality 21, 267–281. 10.1080/1357650X.2015.1127253 26754104

[B12] Oosawa R. Iwasaki R. Suzuki T. Tanabe S. Sugawara K. (2019). Neurophysiological analysis of intermanual transfer in motor learning. Front. Hum. Neurosci. 13, 135. 10.3389/fnhum.2019.00135 31057384 PMC6482209

[B13] Parlow S. E. Kinsbourne M. (1989). Asymmetrical transfer of training between hands: implications for interhemispheric communication in normal brain. Brain Cogn. 11, 98–113. 10.1016/0278-2626(89)90008-0 2789820

[B14] Ruddy K. L. Carson R. G. (2013). Neural pathways mediating cross education of motor function. Front. Hum. Neurosci. 7, 397. 10.3389/fnhum.2013.00397 23908616 PMC3725409

[B15] Sainburg R. L. (2002). Evidence for a dynamic-dominance hypothesis of handedness. Exp. Brain Res. 142, 241–258. 10.1007/s00221-001-0913-8 11807578 PMC10710695

[B16] Sainburg R. L. Schaefer S. Y. Yadav V. (2016). Lateralized motor control processes determine asymmetry of interlimb transfer. Neuroscience 334, 26–38. 10.1016/j.neuroscience.2016.07.043 27491479 PMC5086434

[B17] Sainburg R. L. Wang J. (2002). Interlimb transfer of visuomotor rotations: independence of direction and final position information. Exp. Brain Res. 145, 437–447. 10.1007/s00221-002-1140-7 12172655 PMC10704413

[B18] Scripture E. W. Smith T. L. Brown E. M. (1894). On the education of muscular control and power. Stud. Yale Psychol. Lab. 2, 114–119.

[B19] Serrien D. J. Ivry R. B. Swinnen S. P. (2006). Dynamics of hemispheric specialization and integration in the context of motor control. Nat. Rev. Neurosci. 7, 160–166. 10.1038/nrn1849 16429125

[B20] Shaw P. Greenstein D. Lerch J. Clasen L. Lenroot R. Gogtay N.. (2006). Intellectual ability and cortical development in children and adolescents. Nature 440, 676–679. 10.1038/nature04513 16572172

[B21] Stöckel T. Weigelt M. (2012). Brain lateralisation and motor learning: selective effects of dominant and non-dominant hand practice on the early acquisition of throwing skills. Laterality 17, 18–37. 10.1080/1357650X.2010.524222 21500083

[B22] Stöckel T. Weigelt M. Krug J. (2011). Acquisition of a complex basketball-dribbling task in school children as a function of bilateral practice order. Res. Q. Exerc. Sport 82, 188–197. 10.1080/02701367.2011.10599746 21699098

[B23] Taylor H. G. Heilman K. M. (1980). Left-hemisphere motor dominance in righthanders. Cortex 16, 587–603. 10.1016/S0010-9452(80)80006-2 7226856

[B24] Taylor J. A. Krakauer J. W. Ivry R. B. (2014). Explicit and implicit contributions to learning in a sensorimotor adaptation task. J. Neurosci. 34, 3023–3032. 10.1523/JNEUROSCI.3619-13.2014 24553942 PMC3931506

[B25] Teixeira L. A. Caminha L. Q. (2003). Intermanual transfer of force control is modulated by asymmetry of muscular strength. Exp. Brain Res. 149, 312–319. 10.1007/s00221-002-1363-7 12632233

[B26] Teixeira L. A. Silva M. V. Carvalho M. (2003). Reduction of lateral asymmetries in dribbling: the role of bilateral practice. Laterality 8, 53–65. 10.1080/713754469 15513215

[B27] van Duinen H. Renken R. Maurits N. M. Zijdewind I. (2008). Relation between muscle and brain activity during isometric contractions of the first dorsal interosseus muscle. Hum. Brain Mapp. 29, 281–299. 10.1002/hbm.20388 17394210 PMC6870705

[B28] Wang J. Sainburg R. L. (2004). Interlimb transfer of novel inertial dynamics is asymmetrical. J. Neurophysiol. 92, 349–360. 10.1152/jn.00960.2003 15028745 PMC10709821

[B29] Wang J. Sainburg R. L. (2006). Interlimb transfer of visuomotor rotations depends on handedness. Exp. Brain Res. 175, 223–230. 10.1007/s00221-006-0543-2 16733695 PMC10705045

[B30] Wang Y. Zhao J. Inada H. Négyesi J. Nagatomi R. (2022). Impact of handedness on interlimb transfer depending on the task complexity combined with motor and cognitive skills. Neurosci. Lett. 10, 136775. 10.1016/j.neulet.2022.136775 35817313

[B31] Wang Y. F. Zhao J. Negyesi J. Nagatomi R. (2020). Differences in the magnitude of motor skill acquisition and interlimb transfer between left- and right-handed subjects after short-term unilateral motor skill practice. Tohoku J. Exp. Med. 251, 31–37. 10.1620/tjem.251.31 32434999

[B32] Witkowski M. Bojkowski Ł. Karpowicz K. Konieczny M. Bronikowski M. Tomczak M. (2020). Effectiveness and durability of transfer training in fencing. Int. J. Environ. Res. Public Health 17, 849. 10.3390/ijerph17030849 32013174 PMC7038032

[B33] Witkowski M. Bronikowski M. Nowik A. Tomczak M. Strugarek J. Króliczak G. (2018). Evaluation of the effectiveness of a transfer (interhemispheric) training program in the early stages of fencing training. J. Sports Med. Phys. Fitness 58, 1368–1374. 10.23736/S0022-4707.17.07556-9 28738669

